# Does unemployment contribute to poorer health-related quality of life among Swedish adults?

**DOI:** 10.1186/s12889-019-6825-y

**Published:** 2019-04-29

**Authors:** Fredrik Norström, Anna-Karin Waenerlund, Lars Lindholm, Rebecka Nygren, Klas-Göran Sahlén, Anna Brydsten

**Affiliations:** 1 Department of Epidemiology and Global Health, Umeå University, Umeå, Sweden; 2 Department of Public Health Sciences, Stockholm University, Stockholm, Sweden

**Keywords:** EuroQol 5 dimensions, Labor market, Propensity scores, Quality-adjusted life years

## Abstract

**Background:**

Previous studies have shown that unemployment has negative impacts on various aspects of health. However, little is known about the effect of unemployment on health-related quality of life. Our aim was to examine how unemployment impacts upon health-related quality of life among Swedish adults, and to investigate these effects on population subgroups defined by education level, marital status, previous health, and gender.

**Methods:**

As part of a cross-sectional study, a questionnaire was sent to 2500 randomly selected individuals aged 20 to 64 years living in Sweden in 2016. The questionnaire included the EuroQol 5 dimensions (EQ-5D) instrument and was answered by 967 individuals (39%). Quality-adjusted life year (QALY) scores were derived from the EQ-5D responses. Of the respondents, 113 were unemployed and 724 were employed. We used inverse probability-weighted propensity scores in our analyses to estimate a risk difference. Gender, age, education level, marital status, and previous health were used as covariates in our analyses.

**Results:**

There was a statistically significant lower QALY score by 0.096 points for the unemployed compared to the employed. There were also statistically significant more problems due to unemployment for usual activities (6.6% more), anxiety/depression (23.6% more), and EQ-5D’s Visual Analogue Scale (7.5 point lower score). Grouped analyses indicated a larger negative health effect from becoming unemployed for men, those who are married, and young individuals.

**Conclusions:**

In our study, we show that the health deterioration from unemployment is likely to be large, as our estimated effect implies an almost 10% worse health (in absolute terms) from being unemployed compared to being employed. This further highlights that unemployment is a public health problem that needs more focus. Our study also raises further demands for determining for whom unemployment has the most negative effects and thus suggesting groups of individuals who are in greatest need for labor market measures.

**Electronic supplementary material:**

The online version of this article (10.1186/s12889-019-6825-y) contains supplementary material, which is available to authorized users.

## Background

Unemployment is in general something that has a negative effect on health [1–3], not only at the time of becoming unemployed, but also in a longer time span [4–8]. This relationship has previously been shown for different dimensions of self-assessed, mental and physical health and for depressive symptoms [2, 3]. In previous research, priorities have often been focused on determining the magnitude of the effect at the population level, e.g. through the meta-analyses by McKee-Ryan et al. [1] and by Paul and Moser [3]. However, the study context is highly important to consider because the health consequences from unemployment vary between groups of individuals, e.g. men and women, but also between and within countries and over time [2]. For some groups of individuals, e.g. for Spanish women [9] and for Swedes with only a primary-school education [10], there are even indications of a positive or no effect on health from unemployment. Therefore, policy decisions attempting to reduce unemployment from a health perspective need to consider for whom it is most important to create new job opportunities. Despite a growing literature about the health aspects from becoming unemployed, there is still not much knowledge about how unemployment affects groups of individuals in relation to education level, marital status, previous health, and gender [2].

Despite many health measures being used to study the effect on health from unemployment, studies using health-related quality of life are lacking, and probably the most commonly used instrument in public health research, the EuroQol 5 dimensions (EQ-5D) [11], has as far as we know, not previously been used in unemployment research. The EQ-5D instrument consists of a descriptive system with five questions, a visual analogue scale (EQ-VAS), and a value set. An advantage with the EQ-5D is that its value set, which is derived from responses to the descriptive system, allows it to be translated to so-called Quality Adjusted Life-Year (QALY) scores [12, 13]. The QALY score has two anchor points, 0 (death) and 1 (full health), which makes it possible to compare the magnitude of different public health problems. QALYs also enable comparisons between conducted and potential interventions in a systematic manner, which makes them attractive and commonly used in cost-effectiveness studies of new interventions.

In studies of unemployment and health, unemployment might at least partly be due to poor health, and for this reason one or more variables related to health before unemployment are therefore important as part of the statistical analyses. It is also important to add other potentially confounding variables. In previous research, gender, age, education level, marital status, household income, geographic location, and social network/support have been used most frequently in the statistical models and in presentations of stratified estimates [2]. Gender might be the most important variable to stratify for based on previous research and there are studies that show that women are more affected than men by unemployment as well as studies showing the opposite [2].

Our aim was to examine how unemployment impacts upon health-related quality of life among Swedish adults, and investigate these effects on population subgroups defined by education level, marital status, previous health, and gender.

## Methods

### Study design and participants

A cross-sectional survey consisting of a questionnaire combined with register data from Statistics Sweden was conducted during 2016. From the 5,671,149 individuals who were between 20 and 64 years old and lived in Sweden at the time of the survey, 2500 individuals were selected with simple random sampling and were invited to participate in the study. The questionnaire, which was administered by Statistics Sweden, was sent to the home addresses of the invited individuals on May 2 with two reminders (May 18 and June 1). Participants consented to participate in the study when they returned their questionnaire. There were 967 individuals (39%) who responded to the questionnaire.

The questionnaire consisted of questions related to the participant’s labor market position, health status, health care consumption, and socio-economic and demographic conditions (including education level and marital status). We used self-reported questions related to labor market status to define exposure to unemployment, the EQ-5D as the outcome variable, and gender, age, education level, marital status, and previous health as potentially confounding variables.

Questionnaire responses were scanned and merged with register data that Statistics Sweden administers, and thereafter de-identified, by Statistics Sweden. Register data included, among other things, information about historical unemployment and demographics of the study participants. In the current study, we only used register data to validate the age and gender of the participants. Our choice of variables were consistent with variables in studies similar to ours, with previous health as the only exception [2]. The Regional Ethical Board in Umeå, Sweden, approved the survey.

### Definition of labor market status

The study participants’ labor market status was determined based on the questions: “Which is your main employment” – with ten response alternatives – and “How long have you been unemployed in the last three years?” – with five response alternatives. Those who responded that they had at least six months of unemployment during the last three years were categorized as unemployed (*n* = 113), and those among the other respondents who had responded that their main employment was “gainfully employed” (*n* = 720) or “labor market activity” (*n* = 4) were defined as employed (*n* = 724). Labor market activity refers to a job that is subsided by the state to give the unemployed work experience in order to establish themselves in the labor market. We defined those engaged in labor market activities as employed because we argue that they, in line with what Jahoda has proposed, have a time structure for their waking day, regular contacts with people outside the nuclear family, a purpose of the day transcending their own, and an enforced activity [14]. Thus, their situation is similar to the gainfully employed. The 837 employed and unemployed participants were defined as active in the labor market. The remaining 130 individuals were excluded from our analyses because they had either a different main employment than “gainfully employed” or “labor market activity” or had not responded to this question. There were 52 participants who had experienced unemployment of less than six months, and of these 36 were defined as employed and 16 were excluded.

### Health-related quality of life variables

The EQ-5D was used to measure health-related quality of life [11]. We used the descriptive system with five questions that measure different dimensions of health (mobility, self-care, usual activities (such as work, studies, housework, family, and leisure activities), pain/discomfort, and anxiety/depression) and three response alternatives (corresponding to no, some, or extreme problems). Responses to these questions were translated to QALY scores based on the United Kingdom value set for the EQ-5D, which was derived using linear regression and based on the responses to the EQ-5D descriptive system [12]. The main emphasis in our study was on the QALY scores, but we also present results for the other parts of the EQ-5D instrument, i.e. the dimensions themselves, and EQ-VAS. With the EQ-VAS, respondents valued their current health on a visual analogue scale ranging from 0 to 100. Responses to the EQ-5D dimensions were dichotomized into two groups for analyses of the dimensions themselves, with some and extreme problems combined into one of the two groups, while all three levels were used for QALY calculations.

### Other variables

For gender, man was used as the reference group. We used age as a continuous variable. We also tested age categorized into three age groups (20–34, 35–49, and 50–64), but this did not provide results that seemed to improve the statistical model. Education level was divided into three groups based on the question “What is your highest education?” – 9 years at public school or less was categorized as “primary education”, “secondary education”, and university or college studies was categorized as “university” – with primary education being the reference group. For marital status, the response “living with wife/husband/cohabitant/partner” to the question “How do you live?” was coded as “married” and was used as the reference group, while other responses to the question were defined as “single” and used as the exposure group. Previous health was defined from the question “How was, in general, your health five years ago?”, where the responses “very good” and “fairly good” were defined as “good” and used as the reference group, while the other responses (“fair”, “fairly bad”, and “very bad”) were defined as “poor”.

### Statistics

In our analyses, propensity score weighting was used [15]. Propensity scores were introduced in 1983 by Rosenbaum and Rubin [16], and they correspond to the conditional probability of being assigned to the exposure group based on baseline covariates. A more thorough description and explanation of the use of propensity scores in the current study is available in Norström et al. [6].

We used logistic regression with our potential confounders (gender, age, education level, marital status, and previous health) as covariates in order to estimate the propensity scores. In our study, the propensity score corresponds to the probability of being unemployed given his or her characteristics. Comparisons between an exposed and unexposed individual with the same propensity measure is therefore similar to analyzing exposure in a randomized controlled trial. Using the propensity score approach for observational studies is a quasi-experimental approach.

We used propensity scores weighting, as we favored this approach above matching, and stratification, which are other popular propensity score approaches [17]. We expected the other approaches to perform poorer as they would include fewer unemployed participants because of matching problems with employed individuals. However, there is still a lack of consensus about recommendations on when to use the different approaches. For further discussion about the propensity score approaches, see, for example, Schroeder et al. [17].

In our results, we used the risk difference, which corresponds to the marginal effect of becoming unemployed, with counterfactual arguments. We used an inverse probability weight estimator, as suggested by Lunceford and Davidian [15], to estimate the risk difference

$$ {RD}_{IPW}={\left(\sum \limits_{i=1}^n\frac{X_i}{PS_i}\right)}^{-1}\sum \limits_{i=1}^n\frac{Y_i{X}_i}{PS_i}-{\left(\sum \limits_{i=1}^n\frac{1-{X}_i}{1-{PS}_i}\right)}^{-1}{\sum \limits_{i=1}^n}_i\frac{Y\left(1-{X}_i\right)}{1-{PS}_i}, $$

where *Y* refers to the outcome (health-related quality of life). The marginal effect from this estimator corresponds to the average treatment effect [18]. The standardized difference was calculated, both with and without a weight, to assess the balance of covariates between the employed and unemployed groups for each potential confounder [6, 19].

To be part of our analyses of the descriptive system and the QALY scores, it was required that participants had responded to all variables, including the five health dimensions, with a valid response. Of the 837 individuals who were active in the labor market, 788 were part of our analyses. Of the 49 excluded participants, 14 had not answered at least one of the EQ-5D questions, 14 had no response to education level, 3 had no response to marital status, and 20 had no response to previous health. Two of them had no response to at least two of the variables. Valid responses were also required for all variables for the EQ-VAS analyses. There were 771 valid responses for the EQ-VAS analyses.

Descriptive statistics were used to present the characteristics of the sample, and stratified results were derived for each covariate for the outcome variables. Analyses were carried out for QALY scores, EQ-VAS, and three of the dimensions of the EQ-5D separately as outcome variables. For the first two dimensions of the EQ-5D (mobility and self-care), too few participants reported any problem, and these results were therefore only presented descriptively. Pearson’s χ^2^-test was used to test if the exposure variable (labor market status) was associated with potential confounders. Student’s t-test was used to test differences in age with respect to QALYs between the employed and unemployed.

There are some potential problems with low QALY scores, such as a large gap between QALY scores if responding with some or extreme problems, low QALY scores potentially being related to poor employability, and a low QALY score potentially implying poor health already ahead of unemployment. We therefore performed two different sensitivity analyses. In scenario 1, we chose to exclude those who had answered extreme problems to any of the first three EQ-5D questions (mobility, self-care, and usual activities), and in scenario 2 we excluded participants who had answered extreme problems to any of the EQ-5D questions. Sensitivity analyses were not performed for EQ-VAS.

For logistic regression, it is recommended that the number of individuals of the least occurring event, in our case unemployed, divided by the number of explanatory variables should be at least 10 [20]. This condition was not fulfilled in many of our stratified analyses, which we have indicated in our tables. Also, for other grouped analyses the interpretations should be handled with care due to the small number of unemployed. Interactions between variables were not considered in any of our analyses. We did not experience problems due to collinearity between variables, and hence all candidate variables were kept in the analyses.

R Studio was used for statistical analyses (R Studio, Boston, MA), with its GLM procedure used for logistic regression, where confidence intervals were derived with the profile likelihood [21]. The Bootstrap technique with replacement was used to derive the mean square error from 10,000 replicates. Confidence intervals corresponded to the 2.5 and 97.5% percentiles of the Bootstrap simulations [22]. Based on the Bootstrap simulations, *p*-values were derived. Statistical significance was defined at the 5% level.

## Results

### General characteristics

The proportion of unemployed (12.9%) after removal of missing data was similar to the proportion for the full data set (13.5%). In comparison with the employed, the unemployed reported to a greater extent previous poor health (52% compared to 22%), were more commonly single (48% compared to 22%), and were younger (mean age of 41 compared to 47), and these differences between the groups were statistically significant (Table 1). There was also a statistically significant association between education level and labor market status, with a higher proportion of unemployed than employed having only a primary education.

**Table 1:** Characteristics for the study population

	Employed (*n* = 686)			Unemployed (*n* = 102)		
	n	%		n	%	
Gender						
Man (*n* = 337)	295	43		42	41	
Woman (*n* = 451)	391	57		60	59	
Education level^c^						
Primary education (*n* = 82)	55	8.0		27	26	
Secondary education (*n* = 325)	286	42		39	38	
University (*n* = 381)	345	50		36	35	
Marital status^c^						
Married (*n* = 585)	532	78		53	52	
Single (*n* = 203)	154	22		49	48	
Previous health^a,c^						
Poor (*n* = 198)	149	22		49	52	
Good (*n* = 590)	537	78		53	48	
	mean	median	SD^b^	mean	median	SD^b^
Age^c^	47	48	11	41	39	14

^a^Self-rated health five years ago

^b^Standard deviation

^c^Significance at 5% level using χ^2^ or t-test

### Effect of unemployment on health

The responses to the EQ-5D questions are presented in Table 2. The mean QALY scores were higher among employed than unemployed when no adjustments were made for potentially confounding variables. The propensity scores of the main model gave evidence, through statistical significance in the logistic regression, of a higher likeliness of being unemployed for those with only primary education, those who are single, and those with poor previous health, while the likelihood of unemployment was lower with increased age (Additional file 1: Table S1).

**Table 2:** Responses to EuroQol 5 dimensions

	Employed (n = 686)			Unemployed (n = 102)		
	n	%		n	%	
Mobility						
No problems (*n* = 736)	647	94		89	87	
Some problems (*n* = 52)	39	5.7		13	13	
Extreme problems (*n* = 0)	0	–		0	–	
Self-care						
No problems (*n* = 782)	684	99.7		98	96	
Some problems (n = 4)	0	–		4	3.9	
Extreme problems (n = 2)	2	0.3		0	–	
Usual activities						
No problems (*n* = 717)	639	93		78	76	
Some problems (*n* = 65)	46	6.7		19	19	
Extreme problems (*n* = 6)	1	0.1		5	4.9	
Pain/discomfort						
No problems (*n* = 405)	359	52		46	45	
Some problems (*n* = 354)	310	45		44	43	
Extreme problems (*n* = 29)	17	2.5		12	12	
Anxiety/depression						
No problems (*n* = 478)	446	65		32	31	
Some problems (*n* = 285)	231	34		54	53	
Extreme problems (*n* = 25)	9	1.3		16	16	
	mean	median	SD^b^	mean	median	SD^b^
QALY score^a^	0.84	0.85	0.17	0.68	0.80	0.32
EQ-VAS^c^ (*n* = 771)	79.5	80	15.0	66.0	70	22.8

^a^Quality-adjusted life years score

^b^Standard deviation

^c^For EuroQol Visual Analogue Scale (EQ-VAS), 674 employed and 97 unemployed were included

There was a statistically significant negative effect on QALY scores from becoming unemployed, and the QALY score was 0.096 points lower for the unemployed compared to the employed. Also, for EQ-VAS there was a statistically significant negative effect on health from becoming unemployed (7.5 point lower scores compared to the employed) (Table 3). For the EQ-5D questions, there were statistically significant negative health effects due to unemployment for usual activities (6.6% more estimated to have problems among the unemployed than the employed) and anxiety/depression (23.6% more estimated to have problems among the unemployed than the employed). There was also a greater proportion of unemployed individuals with pain/discomfort problems, but this difference was not statistically significant. For scenario 1 of the sensitivity analyses, the effects differed by at most 0.014, i.e. a marginal difference compared to the main analyses (Table 3). Results for scenario 2 showed different results than the main analysis with some results for the scenario having a substantially smaller negative, and non-significant, effect due to unemployment compared to the main analyses.

**Table 3:** Effect on health-related quality of life from becoming unemployed (*n* = 788)

Health measure	Risk difference^a^	Confidence interval	p	Mean Squared Error
Quality-adjusted life years	−0.096^b^	[−0.158, −0.041]	< 0.001	0.0379
EQ-5D^c^ - Usual activities	0.066^d^	[0.004, 0.140]	0.036	0.0012
EQ-5D^c^ - Pain/discomfort	0.064^d^	[− 0.085, 0.213]	0.395	0.0058
EQ-5D^c^ - Anxiety/depression	0.236^d^	[0.087, 0.385]	0.002	0.0058
EQ-VAS^e^	−7.54^b^	[−12.5, −2.99]	< 0.001	5.840
Sensitivity analysis, scenario 1 (*n* = 780)^f^				
Quality-adjusted life years	−0.084^b^	[−0.145, − 0.029]	0.002	0.0289
EQ-5D - Usual activities	0.053^d^	[−0.009, 0.127]	0.107	0.0012
EQ-5D - Pain/discomfort	0.058^d^	[−0.091, 0.204]	0.449	0.0057
EQ-5D - Anxiety/depression	0.232^d^	[0.083, 0.378]	0.002	0.0057
Sensitivity analysis, scenario 2 (*n* = 741)^g^				
Quality-adjusted life years	−0.028^b^	[− 0.062, 0.006]	0.108	0.0035
EQ-5D - Usual activities	0.013^d^	[− 0.038, 0.076]	0.688	0.0009
EQ-5D - Pain/discomfort	0.032^d^	[− 0.134, 0.193]	0.696	0.0069
EQ-5D - Anxiety/depression	0.211^d^	[0.051, 0.375]	0.008	0.0067

^a^Propensity scores were derived using gender, age, education level, marital status, and previous health for the participants

^b^A risk difference above 0 means less problem with health-related quality of life for unemployed than employed

^c^EuroQol 5 dimensions

^d^A risk difference above 0 means more problem with health-related quality of life for unemployed than employed

^e^EuroQol Visual Analogue Scale

^f^Excluding those with major problems with movement, hygiene, or usual activities

^g^Excluding those with major problems on any level

The balance of the covariates was improved with the propensity scores. The standardized difference ranged from 3.7 to 57% when weights for the QALY score estimates were not applied and from 1.5 to 12% when such weights were applied (Additional file 3: Table S3). The balance was not optimal for gender because the standardized difference was above 10%, the level that according to Austin and Stuart is considered by some experts to be a negligible imbalance [19]. For other variables, the imbalance in observed baseline covariates could be considered as negligible according to the literature.

### Effect of unemployment on health in groups of individuals

Stratified results are presented for QALY score in Table 4, for EQ-VAS in Table 5 and for EQ-5D dimensions in Table 6.

**Table 4:** Stratified results for the effect of unemployment on health on Quality-Adjusted Life-Years (QALY) (n = 788)

	Risk difference^a^	Confidence interval	P-value
Gender			
Man (n = 337)	− 0.083	[− 0.16, − 0.01]	0.023
Woman (n = 451)	−0.108	[−0.21, − 0.02]	0.017
Age			
20–34 years old (*n* = 165)	−0.126	[−0.21, − 0.06]	< 0.001
35–49 years old (*n* = 271)^c^	− 0.112	[− 0.31, 0.04]	0.162
50–64 years old (*n* = 352)^c^	− 0.055	[− 0.15, 0.02]	0.172
Education level			
Primary education (n = 82)^c^	−0.043	[−0.22, 0.09]	0.547
Secondary education (n = 325)^c^	−0.123	[−0.31,0.02]	0.089
University (n = 381)^c^	−0.078	[−0.14, − 0.02]	0.009
Marital status			
Single (n = 203)	−0.022	[−0.13, 0.06]	0.582
Married (n = 585)	−0.109	[−0.19, − 0.04]	< 0.001
Previous health^b^			
Poor (*n* = 198)	−0.244	[−0.37, − 0.12]	< 0.001
Good (*n* = 590)	− 0.041	[− 0.11, 0.02]	0.188

^a^The risk difference presents the mean change in QALY due to unemployment

^b^Self-rated health five years ago

^c^Logistic regression was used with fewer than the recommended 10 outcomes per variable for the least-occurring outcomes

**Table 5:** Stratified results for the effect of unemployment on health on EuroQol 5 dimensions Visual Analogue Scale (*n* = 771)

	Risk difference^a^	Confidence interval	P-value
Gender			
Man (*n* = 329)	− 9.19	[− 17.8, −1.70]	0.018
Woman (*n* = 442)	−6.73	[−14.1, − 0.56]	0.033
Age			
20–34 years old (*n* = 154)^c^	−7.25	[−14.1, − 0.55]	0.034
35–49 years old (*n* = 271)^c^	−11.0	[−27.5, 2.58]	0.108
50–64 years old (*n* = 346)^c^	−3.20	[−9.24, 2.25]	0.258
Education level			
Primary education (n = 77)^c^	−2.29	[−18.7, 16.0]	0.801
Secondary education (*n* = 314)^c^	−10.1	[−21.0, −1.27]	0.023
University (*n* = 380)^c^	−6.92	[−13.5, − 1.27]	0.016
Marital status			
Single (*n* = 194)	−8.47	[−17.0, − 1.77]	0.011
Married (*n* = 577)	−7.64	[−14.1, − 1.72]	0.001
Previous health^b^			
Poor (*n* = 193)	−13.8	[−21.9, −5.92]	0.001
Good (*n* = 578)	−4.71	[−10.1, 0.06]	0.052

^a^The risk difference presents the mean change due to unemployment

^b^Self-rated health five years ago

^c^Logistic regression was used with fewer than the recommended 10 outcomes per variable for the least-occurring outcomes

**Table 6:** Stratified results for the effect of unemployment on health for the EQ-5D dimensions (n = 788)

	Risk difference^a^	Confidence interval	p
Usual activities			
Gender			
Man (n = 337)	0.093	[0.005, 0.207]	0.036
Woman (n = 451)	0.048	[− 0.04, 0.160]	0.312
Age			
20–34 years old (n = 165)	0.124	[0.012, 0.261]	0.027
35–49 years old (n = 271)^c^	0.046	[− 0.072, 0.248]	0.628
50–64 years old (*n* = 352)^c^	0.026	[− 0.036, 0.100]	0.417
Education level			
Primary education (n = 82)^c^	−0.029	[− 0.076, 0.301]	0.282
Secondary education (n = 325)^c^	0.184	[0.023, 0.402]	0.020
University (n = 381)^c^	0.130	[−0.026, 0.352]	0.114
Marital status			
Single (n = 203)	0.038	[− 0.051, 0.148]	0.410
Married (n = 585)	0.091	[0.003, 0.198]	0.042
Previous health^b^			
Poor (n = 198)	0.210	[0.043, 0.377]	0.012
Good (n = 590)	0.007	[−0.040, 0.071]	0.873
Pain/discomfort			
Gender			
Man (n = 337)	0.149	[−0.10, 0.38]	0.218
Woman (n = 451)	0.004	[−0.19, 0.20]	0.976
Age			
20–34 years old (n = 165)	0.088	[−0.09, 0.28]	0.353
35–49 years old (n = 271)^c^	0.030	[−0.27, 0.37]	0.889
50–64 years old (n = 352)^c^	0.020	[−0.22, 0.26]	0.837
Education level			
Primary education (n = 82)^c^	0.111	[−0.08, 0.30]	0.254
Secondary education (n = 325)^c^	−0.046	[−0.29, 0.23]	0.733
University (n = 381)^c^	−0.117	[−0.43, 0.25]	0.550
Marital status			
Single (n = 203)	−0.185	[−0.34, 0.02]	0.069
Married (n = 585)	0.092	[−0.09, 0.27]	0.296
Previous health^b^			
Poor (n = 198)	0.180	[0.01, 0.33]	0.037
Good (n = 590)	0.041	[−0.18, 0.25]	0.703
Anxiety/depression			
Gender			
Man (n = 337)	0.262	[−0.002, 0.54]	0.052
Woman (n = 451)	0.205	[0.01, 0.40]	0.040
Age			
20–34 years old (n = 165)	0.269	[0.08, 0.44]	0.008
35–49 years old (n = 271)^c^	0.176	[−0.18, 0.53]	0.290
50–64 years old (n = 352)^c^	0.168	[−0.04, 0.40]	0.121
Education level			
Primary education (n = 82)^c^	0.261	0.05–0.46	0.016
Secondary education (n = 325)^c^	0.144	−0.09–0.43	0.221
University (n = 381)^c^	0.238	−0.09–0.65	0.148
Marital status			
Single (n = 203)	0.071	−0.17–0.35	0.509
Married (n = 585)	0.280	0.10–0.46	0.002
Previous health^b^			
Poor (n = 198)	0.252	0.09–0.40	0.005
Good (n = 590)	0.210	−0.003–0.42	0.054

^a^The risk difference presents the change in the proportion with health problems due to unemployment

^b^Self-rated health five years ago

^c^Logistic regression was used with fewer than the recommended 10 outcomes per variable for the least-occurring outcomes

For gender, there was a statistically significant negative effect from unemployment on health for both men and women for the QALY score and for the EQ-VAS scale. For anxiety/depression, there was only a statistically significant difference for women. However, the estimated negative effect due to unemployment was greater for men than women and was almost significant. For all EQ-5D questions, there was a greater negative effect for men than women related to unemployment, but it was only for usual activities that men had a statistically significant effect. For the sensitivity analyses, there was no statistical evidence for a negative effect on QALY score for either men or women (Additional file 2: Table S2). Interestingly, the QALY score, which is a summary measure of the five EQ-5D questions, for men was lower than for women in the main analysis despite the results on any of the EQ-5D questions showing a larger, though not in all cases statistically significant, negative effect for men than for women due to unemployment. In the second scenario for the sensitivity analysis, however, the estimate of the negative effect due to unemployment was greater even for the QALY scores for men.

For results on age, there was a statistically significant negative effect on health due to unemployment for the youngest age group (20–34 years old) for the QALY score, EQ-VAS, and the anxiety/depression dimension, but not for the other EQ-5D dimensions. For the other two age groups, the estimated effects were mostly smaller than for the 20–34 year olds but were not statistically significant. For these two age groups, the number of unemployed was too few to fulfill the criterion for the number of events per variable. For education level, no group fulfilled this criterion. For education level, the negative health effect from becoming unemployed was statistically significant for QALY score, EQ-VAS, and the anxiety/depression dimension for university studies, and for secondary education the negative health effect from becoming unemployed was statistically significant for EQ-VAS and usual activities.

For marital status, becoming unemployed was negative for health for those who were married, with statistical significances for QALY score, EQ-VAS, usual activities, and anxiety/depression, while there were no evidence of negative effects for participants who were single, except for EQ-VAS. Previously poor health was a negative factor for becoming unemployed, with a statistically significant estimated QALY decrement of 0.24 compared to being employed, and there was a statistical significance for all of the presented EQ-5D dimensions. For those with good previous health, there were no statistically significant differences. However, the estimated negative effect was rather large and close to significant for the anxiety/depression dimension (*p* = 0.054).

Sensitivity analyses for other variables than gender showed similar results in most cases (Additional file 2: Table S2). It was mainly results for the second scenario that had inconsistent results, e.g. no significance for QALY or usual activities for the youngest if they became unemployed. Usual activities for single persons and women even showed signs of an improved health if becoming unemployed.

## Discussion

In current study we have explored the impact on health-related quality of life among Swedish adults on unemployment. We show that unemployment is strongly related to a poorer health-related quality of life. The magnitude of the effect is large, with an absolute loss of QALY of 10% from unemployment according to our estimate. The effect on QALY is mainly explained by an increase in problems with anxiety/depression due to unemployment. In our study, 24% more of the unemployed than the employed had problems with anxiety/depression, despite there being as many as 35% of the employed having at least some problem. Also, for usual activities unemployment was shown to affect health negatively, while evidence of an increase in problems with pain/discomfort could not be statistically supported, though these were numerically more common among the unemployed than employed. For grouped analyses, after becoming unemployed married individuals, young individuals, and those who already had poorer health before their unemployment showed greater problems than people did in general.

That unemployment is negative for health has been shown in most previous studies [2, 3], and our study is well in line with these studies. Our study uses QALYs, which through its construction makes it possible to present the magnitude of health effects in both a comparable way with other public health problems and in terms of health-related quality of life [13]. For instance would our estimated effect of 0.096 mean that 9.1 years of employed years would be valued above living 10 years as unemployed. Other studies, such as meta-analyses have presented effect sizes [1, 3], but also effect sizes are difficult to translate in a similar way as QALYs.

It is interesting that it is mainly due to feeling anxious and/or depressed that unemployment causes significant problems in our study. In previous research on unemployment and health, the health measures and the health dimension in focus have varied to a large degree [2]. In the meta-analysis by Paul and Moser, which was published almost 10 years ago [3], effect sizes were presented for different aspects of mental health. Most of these measures showed a similarly sized negative health effect due to unemployment. It is thus difficult to draw firm conclusions about which health aspects are mainly affected by unemployment based on the literature. That our results clearly indicate more extensive problems related to anxiety/depression than for pain/discomfort might be explained by the fact that unemployment has both economic and social consequences. At the time of unemployment, some unemployed might have reduced their bodily burden after becoming unemployed and thus had lower problems with work-related pain/discomfort. Thus, pain/discomfort might be related to more of a problem than our study suggests, and it is advisable that longitudinal and qualitative studies investigate these health problems more in depth in relation to unemployment.

The results we present on the group level indicate, despite a higher estimated QALY for women, that health-related quality of life might be more negatively affected in men than in women by unemployment. Previous results have shown diverging results in a global perspective, as well as in Swedish studies [2]. Among previous Swedish studies, those using the General Health Questionnaire, which focuses on psychological symptoms, including anxiety, have indicated more problems due to unemployment for men than for women [23, 24], while those studies that have shown more problems for unemployed women than men have mainly used a question about self-rated health with 3 or 5 responses [6, 10, 25–27]. Thus, our results that indicate that men are more affected by problems with anxiety and depression than women when they become unemployed are in line with previous studies. It can be speculated that unemployment causes different health consequences for men and women, highlighting the need for looking at health in a broader view for determining who is most strongly affected by unemployment among men and women, but also for other groups of individuals. More research is needed to better understand differences in unemployment experiences for men and women because our study does not provide strong enough evidence to draw firm conclusions.

Our study suggests more problems for young people than others due to unemployment in the short-term perspective. Previous studies have shown varied results from the short-term perspective [2], and it is therefore difficult to draw firm conclusions. It has also been shown that there is a long-term effect on health from youth unemployment [4, 7, 8]. Even if the short-term negative health aspects might not be larger for the younger age groups, still, from the full time spectrum, the largest health consequences are among the youngest, at least if being long-term unemployed. Also, long-term consequences of unemployment have been shown for middle-aged persons despite being re-employed [6].

Few previous studies have presented results for different education levels [2]. Our study could not add much to this knowledge because the numbers of unemployed in the three education groups were too few for stable results. Our study suggests that married people who become unemployed fare worse than single people who become unemployed. Also for this group, the evidence base is weak mainly because results are rarely presented on the group level for marital status.

It is difficult to get good estimates for the effect on health from becoming unemployed, and this is mainly due to problems with health selection. In a cross-sectional study design such as ours, this is an even bigger hurdle to overcome than in the recommended longitudinal studies, which also face problems with showing causality. To improve the estimates, we asked respondents about their previous health status in order to get an idea about the contribution to health deterioration that is from the unemployment episode. Interestingly, despite expressing poor previous health, which was not to a greater extent likely to be related to unemployment, health was more affected for these individuals than for those who had good health before their unemployment. Targeting people with already poor health for labor market measures might therefore be even more important than previously considered, at least in the Swedish context. To our knowledge it is rare that results are presented stratified on a proxy for health before unemployment, such as in our case health 5 years prior to participation in the study. Our study focus is on the short-term perspective. A previous Swedish study, which focused on the long-term perspective of being unemployed, showed a similar long-term effect for those with good and poor health when becoming unemployed [6], thus, also indicating that those with poor health already at the time of unemployment were being affected by unemployment.

As already mentioned, our study has the limitation of being a cross-sectional study. In our study, we had a low response rate which also makes it difficult to draw firm conclusions. However, the number of responders was still large and conclusions for at least the group as a whole should therefore be valid. We defined unemployment as at least six months of unemployment during the past three years. Using current unemployment would have meant too few cases, and it would not have guaranteed that the person had an unemployment episode that would have meant a problem for him/her. Our definition of unemployment is common in similar previous studies, and we believe that it is a good way of capturing the exposure of being unemployed in both the short and long-term perspective.

A strength with our study is that it is the first to study health-related quality of life extensively. Our results on the group level might be weak, but they still provide important information that can be used by decision makers. They also provide important information to be used for future studies.

In our sensitivity analysis, we could show that those who responded as having extreme problems affected the results for gender. For the first scenario we had only small differences with our main scenario where we did not censor extreme responses. For our second scenario, we found some interesting differences, but because these depended on the fourth and fifth dimension where we had a large proportion of individuals, we think that this scenario should be seen as more of an illustration of how sensitive the results are for these responses, and not as a sign that the results from the main analysis are misleading. Still, our results highlight that the EQ-5D should rather be seen as an indicator of health and not as a measure that can produce a fine-tuned picture of an individual’s health status. In our view, no other set of questions than the EQ-5D, which measures self-assessed health, can more precisely estimate health-related quality of life on a scale from 0 to 1, so the EQ-5D should still be strongly advisable to use.

We used the United Kingdom value set for EQ-5D for our analyses [12]. There is a recent Swedish value set for EQ-5D, but it has rarely been used and this makes it difficult to compare QALYs with other populations [28], which is important from a health economics perspective. Our results could therefore be biased due to being based on a non-Swedish population. The United Kingdom value set for EQ-5D is over 20 years old, which might also bias results. However, we find no reason to believe that this would have more than a marginal effect on our findings.

In our study, we would have liked to have split the employed into permanent and temporary employees. However, our small sample size did not allow for such a discrimination of the respondents. Splitting the group of employed might have resulted in a larger gap between the permanently employed and unemployed than we observed in our study, which would, if true, have further emphasized the need for permanent positions instead of unemployment. The debate about stable positions in relation to non-stable positions is a hot topic politically, at least in Sweden, and there is evidence suggesting that job insecurity is as bad for health as unemployment. [29] Knowing more about this is important, but this was outside the scope of the present study.

In our study, we present a QALY difference of 0.096 between the unemployed and employed. As previously touched upon in the discussion, the interpretation of this QALY difference from the health economics theory is that 9.1 years of employment should be valued above 10 years of unemployment, i.e. that it is better to live 9% less lifetime if unemployment can be avoided. This highlights the importance of policy makers prioritizing from the public health perspective rather than from an economic growth perspective. However, the latter tends to dominate discussions about lowering unemployment. We might have overestimated the negative health effect of unemployment, but even so the effect is nevertheless likely to be negligible. In line with a previous review [2], we recommend that more focus should be put on group-wise analyses in order to determine for whom it is most important to prioritize efforts to avoid unemployment, both in regard to political priorities and for future research.

An added value with our study is that, besides increased knowledge about how unemployment relates to health-related quality of life, our results can be used for health economic studies because QALYs are the most common measure used in cost-effectiveness analyses. We also intend to use our results for a future health economic evaluation where we study the cost-effectiveness of increased staffing within home care.

Our study was performed in Sweden. The results are likely to be representative for other countries, e.g. other Nordic countries, that have a similar labor market. Our results should add valuable information also for other European countries even if their labor market system more or less differ from the Swedish.

## Conclusions

In our study, we show that the health deterioration from unemployment is likely to be large, as our estimated effect implies almost 10% poorer health-related quality of life (in absolute terms) from being unemployed compared to being employed, and the problems are most apparent for the anxiety/depression scale of the EQ-5D instrument. Our results show, just like previous research, that unemployment hits groups of individuals differently, and measures directed to specific groups of individuals are therefore recommended. For future research, it is important to put more emphasis on groups of individuals to get a better basis for prioritizations within labor market measures in order to lower the public health impact of unemployment.

## Additional files

Additional file 1: **Table S1.** Logreg coefficients prop score. (DOCX 13 kb)

Additional file 2: **Table S2.** Sensitivity analyses. (DOCX 20 kb)

Additional file 3: **Table S3.** Covariate balancing. (DOCX 16 kb)

## Abbreviations

EQ-5D: EuroQol 5 dimensions
EQ-VAS: EuroQol Visual Analogue Scale
QALY: Quality-Adjusted Life-Year
