# Supplementary material for: Does unemployment contribute to poorer health-related quality of life among Swedish adults?
Source: BMC Public Health. 2019 Apr 29;19:457. doi: 10.1186/s12889-019-6825-y (PMC6489216; doi:10.1186/s12889-019-6825-y)
Supplement: Supplementary file 1 — Table S1. Logreg coefficients prop score. (DOCX 13 kb) [file 12889_2019_6825_MOESM1_ESM.docx]

**Additional file 1**

**Table S1.** Results from logistic regression for determining propensity scores for estimates of quality-adjusted life year scores (n = 788).

| *Variable* | *Odds ratio* | Confidence interval |
| --- | --- | --- |
| **Gender** | 1.02 | 0.64–1.65 |
| **Age** | 0.96 | 0.94–0.98 |
| **Education** |  |  |
| *Primary education (n = 82)* | 6.02 | 3.14–11.6 |
| *Secondary education (n = 325)* | 1.16 | 0.69–1.93 |
| **Marital status** | 2.26 | 1.41–3.63 |
| **Previous health** | 2.94 | 1.85–4.68 |

The odds ratio represents the increased odds of being unemployed based on exposure. Reference values are man, university, partner, and good previous health. Age is a continuous variable.
