# Supplementary material for: Does unemployment contribute to poorer health-related quality of life among Swedish adults?
Source: BMC Public Health. 2019 Apr 29;19:457. doi: 10.1186/s12889-019-6825-y (PMC6489216; doi:10.1186/s12889-019-6825-y)
Supplement: Supplementary file 2 — Table S2. Sensitivity analyses. (DOCX 20 kb) [file 12889_2019_6825_MOESM2_ESM.docx]

**Additional file 2**

**Table S2.** Stratified results for the effect of unemployment, sensitivity analyses

|  | **Scenario** | | **QALY^a^** | | **EQ-5D^b^**  *Usual activities* | | **EQ-5D^b^**  *Pain /*  *Discomfort* | | | **EQ-5D^b^** *Anxiety /*  *Depression* | | |
| --- | --- | --- | --- | --- | --- | --- | --- | --- | --- | --- | --- | --- |
| **Gender** |  |  | |  | |  | | |  | | |  |
| *Man* | *M^c^* | −0.083* | | 0.093* | | 0.149 | | | 0.262 | | |  |
|  | *1^d^* | −0.064* | | 0.080 | | 0.142 | | | 0.257 | | |  |
|  | *2^e^* | −0.049 | | 0.084 | | 0.137 | | | 0.251 | | |  |
|  |  |  | |  | |  | | |  | | |  |
| *Woman* | *M* | −0.108* | | 0.048 | | 0.004 | | | 0.205* | | |  |
|  | *1* | −0.100* | | 0.034 | | −0.000 | | | 0.200* | | |  |
|  | *2* | −0.009 | | −0.051* | | −0.040 | | | 0.147 | | |  |
| **Age** |  |  | |  | |  | |  | | |  |  |
| *20–34 years old* | *M* | −0.126* | | 0.124* | | 0.088 | | | 0.269* | | |  |
|  | *1* | −0.101* | | 0.085 | | 0.080 | | | 0.266* | | |  |
|  | *2* | −0.045 | | 0.021 | | 0.087 | | | 0.247* | | |  |
| *35–49 years old* | *M* | −0.112 | | 0.046 | | 0.030 | | | 0.176 | | |  |
|  | *1* | −0.114 | | 0.047 | | 0.031 | | | 0.179 | | |  |
|  | *2* | −0.015 | | −0.026 | | −0.030 | | | 0.182 | | |  |
| *50–64 years old* | *M* | −0.055 | | 0.026 | | 0.020 | | | 0.168 | | |  |
|  | *1* | −0.036 | | 0.015 | | 0.002 | | | 0.145 | | |  |
|  | *2* | 0.004 | | −0.006 | | −0.039 | | | 0.106 | | |  |
| **Education level** |  |  | |  | |  | |  | | |  |  |
| *Primary education* | *M* | −0.043 | | 0.130 | | −0.117 | | | 0.238 | | |  |
|  | *1* | −0.036 | | 0.119 | | −0.122 | | | 0.235 | | |  |
|  | *2* | 0.001 | | 0.130 | | −0.172 | | | 0.226 | | |  |
| *Secondary education* | *M* | −0.123 | | 0.184* | | −0.046 | | | 0.144 | | |  |
|  | *1* | −0.094 | | 0.166* | | −0.058 | | | 0.133 | | |  |
|  | *2* | 0.016 | | 0.020 | | −0.138 | | | 0.029 | | |  |
| *University* | *M* | −0.078* | | −0.029 | | 0.111 | | | 0.261* | | |  |
|  | *1* | −0.080* | | −0.036 | | 0.109 | | | 0.264* | | |  |
|  | *2* | −0.046* | | −0.029 | | 0.128 | | | 0.273* | | |  |
| **Marital status** |  |  | |  | |  | | |  | | |  |
| *Single* | *M* | −0.022 | | 0.038 | | −0.185 | | | 0.071 | | |  |
|  | *1* | −0.003 | | 0.018 | | −0.192 | | | 0.070 | | |  |
|  | *2* | 0.029 | | −0.053* | | −0.182 | | | 0.089 | | |  |
| *Married* | *M* | −0.109* | | 0.091* | | 0.092 | | | 0.280* | | |  |
|  | *1* | −0.093* | | 0.077 | | 0.082 | | | 0.276* | | |  |
|  | *2* | −0.042* | | 0.048 | | 0.065 | | | 0.255* | | |  |
| **Previous health^f^** |  |  | |  | |  | | |  | | |  |
| *Poor* | *M* | −0.244* | | 0.210* | | 0.180* | | | 0.252* | | |  |
|  | *1* | −0.188* | | 0.146 | | 0.159 | | | 0.233* | | |  |
|  | *2* | −0.034 | | 0.026 | | 0.056 | | | 0.234 | | |  |
| *Good* | *M* | −0.041 | | 0.007 | | 0.041 | | | 0.210 | | |  |
|  | *1* | −0.042 | | 0.008 | | 0.042 | | | 0.211 | | |  |
|  | *2* | −0.029 | | −0.002 | | 0.047 | | | 0.203 | | |  |

^a^ QALY = Quality-Adjusted Life Year score. A risk difference below 0 means an improved quality-adjusted life year score from unemployment.
^b^ EuroQol 5 dimensions. A risk difference above 0 means that unemployment increases the proportion with problems.

^c^ results when sensitivity analysis was not applied (n = 796).

^d^ excluding those with major problems with movement, hygiene, or usual activities (n = 780).

^e^ excluding those with major problems on any level (n = 741).

^f^ Self-rated health five years before questionnaire response.

* p < 0.05
