# Supplementary material for: Does unemployment contribute to poorer health-related quality of life among Swedish adults?
Source: BMC Public Health. 2019 Apr 29;19:457. doi: 10.1186/s12889-019-6825-y (PMC6489216; doi:10.1186/s12889-019-6825-y)
Supplement: Supplementary file 3 — Table S3. Covariate balancing. (DOCX 16 kb) [file 12889_2019_6825_MOESM3_ESM.docx]

**Additional file 3**

**Table S3.** Diagnostics of the inverse probably weights for the reduced model.

|  | **Unweighted^a^** | | | **Weighted^b^** | | | |
| --- | --- | --- | --- | --- | --- | --- | --- |
|  | *Absolute difference^c^* | *Standard deviation^d^* | *Standardized difference^e^* | *Absolute difference^c^* | *Standard deviation^d^* | *Standardized difference^e^* |  |
| **Gender** | 0.018 | 0.494 | 3.7% | 0.059 | 0.499 | 11.8% |  |
| **Age** | 5.79 | 12.9 | 44.8% | 0.558 | 12.3 | 4.5% |  |
| **Education level** |  |  |  |  |  |  |  |
| *Primary education* | 0.185 | 0.366 | 50.4% | 0.015 | 0.301 | 4.8% |  |
| *Secondary education* | 0.035 | 0.489 | 7.1% | 0.024 | 0.491 | 4.9% |  |
| *University* | 0.150 | 0.489 | 30.7% | 0.039 | 0.502 | 7.7% |  |
| **Marital status** | 0.256 | 0.460 | 55.6% | 0.031 | 0.422 | 7.4% |  |
| **Previous health** | 0.263 | 0.458 | 57.4% | 0.007 | 0.436 | 1.5% |  |

^a^ Proportions and mean values in unweighted samples are available in Table 1.
^b^ Estimates after inverse probability weights based on the propensity scores have been applied to balance the groups.
^c^ The estimated absolute difference between unemployed and employed for the variable.
^d^ The standard deviation pools the unemployed (“treatment”) and employed (“control”) samples.
^e^ The absolute value of the standardized difference is presented in %.
